# Effect of Different Extraction Techniques on Phenolic Profile and Phytochemical Potential of *Gymnema inodorum* Leaf Extract

**DOI:** 10.3390/molecules29225475

**Published:** 2024-11-20

**Authors:** Muhammad Hassnain Haideri, Titi Phanjaroen, Wiritphon Khiaolaongam, Thanarat Boonchalaem, Jiraporn Laoung-on, Supakit Chaipoot, Surat Hongsibsong, Kongsak Boonyapranai, Sakaewan Ounjaijean

**Affiliations:** 1 School of Health Sciences Research, Research Institute for Health Sciences, Chiang Mai University, Chiang Mai 50200, Thailand; muhammadhassnain_h@cmu.ac.th (M.H.H.); titi_p@cmu.ac.th (T.P.); wiritphon_k@cmu.ac.th (W.K.); thanarat_boon@cmu.ac.th (T.B.); surat.hongsibsong@cmu.ac.th (S.H.); 2 Multidisciplinary Research Institute, Chiang Mai University, Chiang Mai 50200, Thailand; jiraporn.l@cmu.ac.th (J.L.-o.); supakit.ch@cmu.ac.th (S.C.); 3 Center for Non-Infectious Diseases and Environmental Health Sciences, Research Institute for Health Sciences, Chiang Mai University, Chiang Mai 50200, Thailand; kongsak.b@cmu.ac.th

**Keywords:** *Gymnema inodorum*, antioxidant activity, phenolic profile, phytochemicals, extraction techniques

## Abstract

The therapeutic potential of plant extracts has attracted significant interest, especially regarding indigenous species with health-promoting properties. *Gymnema inodorum*, native to Northern Thailand, is recognized for its rich phytochemical profile; however, the impact of various extraction techniques on its phenolic composition and bioactivity remains underexplored. Optimizing extraction methods is essential to enhance the pharmacological efficacy of this plant’s bioactive compounds. This study investigated the influence of four extraction methods—ethanol maceration, ethanol reflux, aqueous decoction, and microwave-assisted extraction—on the bioactive profile of *G. inodorum* leaves, with a focus on the phenolic content and biological activities. Antioxidant activities were evaluated using DPPH, ABTS, and FRAP assays, while the total phenolic and flavonoid contents were quantified by colorimetric methods. High-Performance Liquid Chromatography (HPLC) quantified gymnemic acid and key phenolic compounds. Among the methods, ethanol reflux yielded the highest antioxidant activities (DPPH and ABTS scavenging), with a total phenolic content of 82.54 mg GAE/g and flavonoid content of 31.90 mg QE/g. HPLC analysis identified sinapic acid, myricetin, and p-hydroxybenzoic acid as major phenolics. Furthermore, the ethanol reflux extract displayed potent anti-diabetic activity, with IC_50_ values of 13.36 mg/mL for α-amylase and 7.39 mg/mL for α-glucosidase, as well as strong anti-inflammatory activity (IC_50_ of 1.6 mg/mL) and acetylcholinesterase inhibition (IC_50_ of 1.2 mg/mL). These findings suggest that ethanol reflux extraction is a highly effective method for producing bioactive-rich *G. inodorum* extracts, with substantial pharmacological potential for developing herbal remedies and nutraceuticals, particularly in enhancing therapeutic approaches for diabetes and other health-related conditions.

## 1. Introduction

The plant belongs to the Apocynaceae family and includes the genus *Gymnema*, which is widespread in tropical and subtropical areas across Asia and Africa, comprising over 50 species listed [1]. *Gymnema inodorum*, locally known as Chiang-da in Northern Thailand [2], is utilized as both a culinary vegetable and medicinal herb. Traditionally, Chiang-da is mainly used as a vegetable for cooking and as a key botanical ingredient in various dishes that undergo essential preparation methods like boiling and stir-frying. It is also air-dried for tea production, especially in the countryside. *G. inodorum* has been devoted to the management of diabetes, obesity, and metabolic diseases with its potential in controlling blood sugar levels and insulin-mimetic activity [3]. Notably, *G. inodorum* has garnered attention for its bioactive compounds, particularly gymnemic acids, which inhibit glucose absorption [4].

While *Gymnema sylvestre*—a species similar to *G. inodorum*—has been extensively studied for its phytochemical constituent as well as its therapeutic potential [5]; the latter remains underexplored despite its traditional use in the same regions. These plants contain significant phytoconstituents, including triterpene saponins (such as gymnemic acids), gymnemasaponins, and polyphenols. Recent studies have further highlighted the plant’s bioactive compounds, particularly gymnemic acid, which is known for its ability to inhibit glucose absorption, making the *Gymnema* plant a promising natural treatment for diabetes [6]. In addition, *G. inodorum* leaf extracts have been reported to possess anti-fungal [7] and antioxidant properties [8], attributed to compounds like ginsenosides.

Recent studies highlight the plant’s antioxidant potential, primarily attributed to its high content of phenolic compounds, including flavonoids, quercetin, and kaempferol [9]. These compounds contribute to its significant free radical scavenging activities, providing protection against oxidative stress and related diseases such as cardiovascular diseases [10,11].

The non-toxic and eco-friendly extraction of these phenolic compounds enhances their appeal for therapeutic applications, showcasing a broad spectrum of biological activities, including anti-inflammatory and anti-cancer effects [12]. Therefore, exploring the phenolic profile and antioxidant potential of *G. inodorum* is imperative to understand its therapeutic potential better.

Given the increasing interest in using natural products to manage chronic conditions, it is critical to further explore the bioactive potential of *G. inodorum.* Specifically, understanding how different extraction methods affect the yield and efficacy of its bioactive compounds is essential. Optimizing extraction parameters is crucial for enhancing the yield and efficacy of bioactive compounds. Response surface methodology (RSM) has effectively optimized conditions such as temperature, time, and solvent ratios for various plant materials. Additionally, ethanol-based extraction techniques have been shown to more effectively isolate phenolic compounds, which possess stronger antioxidant properties than water-based methods [13].

The screening, separation, and quantification of phytochemicals in medicinal plants have greatly advanced, unveiling the benefits of ancient herbal remedies. Traditional medicine has been effective against oxidative stress-related diseases. Extensive research into natural compounds has identified numerous bioactive compounds such as alkaloids, steroids, tannins, glycosides, oils, resins, phenols, terpenoids, and flavonoids, which serve as crucial pharmacological agents [14]. Comparative studies on other medicinal plants have shown that varying extraction techniques significantly influence the yield and biological activity of phytochemicals [15], and a similar approach may be vital for *G. inodorum*. The choice of solvent significantly influences the extraction efficiency and the preservation of heat-sensitive components, underscoring the need for diverse extraction techniques to maximize bioactive compound yields [16].

Given the limited research on the effects of extraction techniques on the polyphenols of *G. inodorum* and their biological activities, this study aims to compare the effectiveness of different extraction techniques in optimizing the bioactive compounds and therapeutic potential of *G. inodorum* leaf. It focuses on the identification and quantification of phenolic compounds and gymnemic acid, along with evaluating their antioxidant, anti-inflammatory, and enzyme-inhibitory properties.

## 2. Results

The extraction yield, total phenolic content (TPC), total flavonoid content (TFC), gymnemic acid concentration, quantification of the phenolic profile of *Gymnema inodorum* extracts, antioxidant activities (ABTS, DPPH, and FRAP assay), enzyme inhibitory activities (α-amylase, α-glucosidase, and acetylcholinesterase enzyme (AChE)), anti-inflammatory activity, and cytotoxicity to macrophage cell line obtained by different extraction methods (aqueous decoction, aqueous microwave-assisted extraction, ethanolic maceration, and ethanolic reflux extraction) were evaluated, linking back to our hypothesis that extraction methods significantly influence these outcomes.

### 2.1. Extraction Yield and Phytochemical Contents

As outlined earlier, the ethanol reflux method offers significant methodological advantages. The results presented in Table 1 demonstrate that this method achieved a yield of 20.12% and the highest total phenolic content, measuring 82.54 ± 0.07 mg GAE/g, compared to other methods. These findings validate ethanol reflux as the most effective extraction technique for isolating bioactive compounds. For water-based extractions, both the decoction and microwave-assisted extraction methods resulted in similarly high percentages of yield (28.00% and 27.18%, respectively) and total phenolic content (54.88 ± 0.09 mg GAE/g and 55.49 ± 0.22 mg GAE/g, respectively). This indicates a strong correlation between the efficacy of the reflux method and the release of phenolic compounds, suggesting that the process enhances solvent interaction with plant material, resulting in more efficient extraction.

The analysis of total flavonoid content (TFC) and gymnemic acid concentration in the *G. inodorum* extracts revealed that ethanolic maceration yielded the highest TFC (35.80 ± 0.01 mg QE/g), followed by ethanolic reflux (31.90 ± 0.03 mg QE/g). In contrast, the highest concentration of gymnemic acid was observed in the ethanolic reflux extract (8.24 ± 0.65 mg/g), highlighting the effectiveness of ethanolic extraction methods in isolating active compounds.

### 2.2. Identification of Phenolic Profile and Quantification of Phenolic Compounds

**Figure 1:**
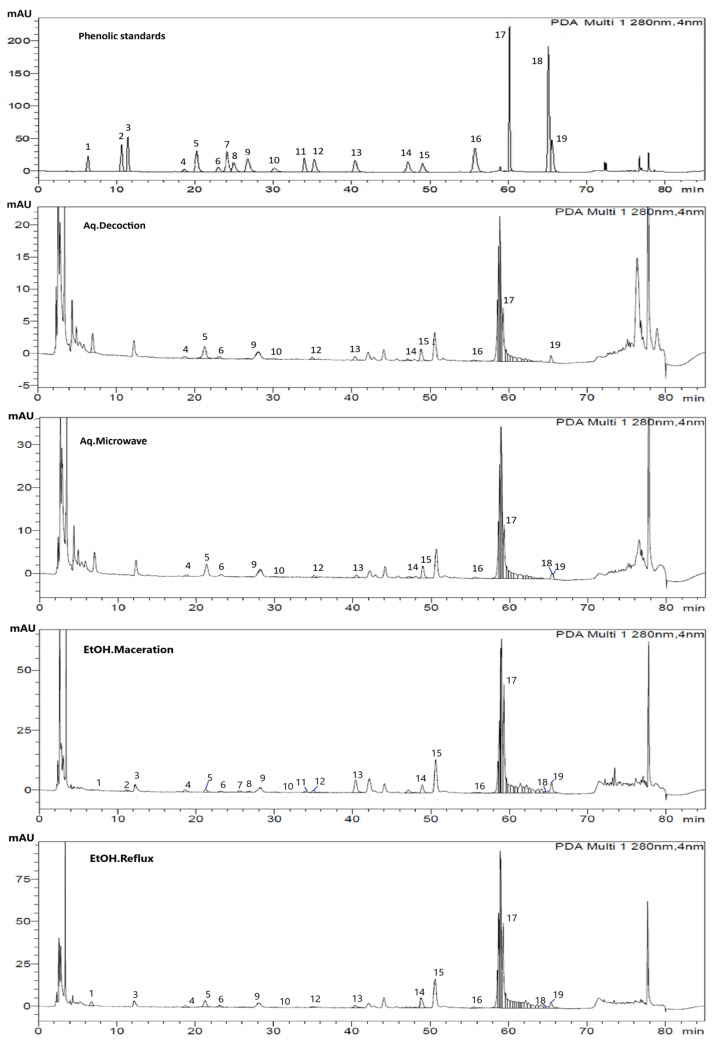
HPLC Chromatograms of phenolic standards and various *G. inodorum* leaf extracts. Compounds were identified based on their retention times and characteristic UV absorbance spectra compared to those of authentic standards. Peak are indicated as follows: (1) gallic acid, (2) Theobromine, (3) Protocatechuic acid, (4) p-Hydroxybenzoic acid, (5) catechin, (6) chlorogenic acid, (7) Caffeine, (8) Vanillic acid, (9) caffeic acid, (10) Syringic acid, (11) epicatechin, (12) Vanillin, (13) p-Coumaric acid, (14) ferulic acid, (15) sinapic acid, (16) rutin, (17) myricetin, (18) quercetin, and (19) Trans-cinnamic acid. Detection was performed at 280 nm.

The quantification of phenolic compounds in the *G. inodorum* extracts using the HPLC technique revealed a distinct variation across the four extraction methods. For the ethanolic reflux extract, 15 out of the 19 phenolic standards were detected. Sinapic acid (1.29 ± 0.01 mg/g) and myricetin (0.93 ± 0.01 mg/g) were the most abundant, while compounds such as p-hydroxybenzoic acid and chlorogenic acid also contributed substantially to its phenolic profile. Antioxidant-related compounds, including gallic acid, catechin, and quercetin, were present in appreciable amounts, potentially enhancing the extract’s bioactivity. Among the extraction methods, the ethanolic reflux approach yielded the highest total phenolic content (3.605 ± 0.013 mg/g), significantly outperforming ethanol maceration (3.104 ± 0.011 mg/g), aqueous microwave-assisted extraction (1.426 ± 0.004 mg/g), and aqueous decoction (0.986 ± 0.003 mg/g). This comprehensive profile suggests that ethanol reflux extraction is particularly effective in maximizing both the yield and diversity of bioactive phenolic compounds in *G. inodorum*, supporting its potential as a potent source of natural antioxidants. Detailed quantitative data on individual phenolic compounds are presented in Table 2, calculated to correspond with the chromatographic profile of the reference standards shown in Figure 1.

### 2.3. Antioxidant Activities of Extracts of G. inodorum

**Figure 2:**
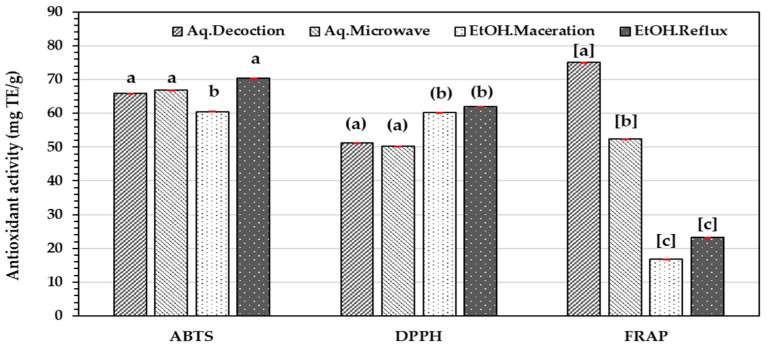
Antioxidant activities of *G. inodorum* leaf extract obtained from various extraction methods determined by different antioxidant assays: ABTS radical scavenging activity, DPPH radical scavenging activity, and Ferric-reducing antioxidant power (FRAP). The statistical significance was determined using one-way ANOVA, followed by Duncan’s multiple range test at *p* < 0.05 and represented by lowercase letters (a, b, or c). The variables with the same letters are not statistically significant. Data are expressed as mean ± SD (*n* = 3).

The antioxidant activity of the leaf extract of *G. inodorum* derived from different extraction methods was assessed using DPPH, ABTS, and FRAP assays, expressed as mg Trolox equivalent (TE) per gram of extract. The ethanolic reflux extract exhibited the highest antioxidant activities in ABTS (70.33 ± 0.00 mg TE/g) and DPPH (61.94 ± 0.01 mg TE/g) assays, while the aqueous decoction showed the most robust FRAP activity (75.10 ± 0.00 mg TE/g). These results indicated that different extraction methods influence the antioxidant capacity of the extracts (Figure 2).

### 2.4. α-Glucosidase and α-Amylase Inhibitory Activities

The result shown in Table 3 demonstrated that ethanolic reflux exhibited the strongest inhibitory effect on key enzymes related to type 2 diabetes, with IC_50_ values of 7.398 mg/mL for α-glucosidase and 13.362 mg/mL for α-amylase, suggesting potential anti-diabetic activity. In contrast, the ethanolic maceration extract exhibited the weakest inhibition of α-amylase, with an IC_50_ of 158.371 mg/mL, highlighting significant differences in enzyme inhibition based on the extraction method. Acarbose, a standard anti-diabetic drug, served as a positive control with IC_50_ values of 5.534 mg/mL for α-glucosidase and 0.999 mg/mL for α-amylase.

### 2.5. Acetylcholinesterase Inhibitory and Anti-Inflammatory Activities

A summary of acetylcholinesterase inhibition (AChEI) by *G. inodorum* leaf extracts from the different extraction methods used in this study is provided in Table 4. The AChEI activity of each extract is indicated by its IC_50_ value. The extract from ethanol reflux extraction demonstrated the highest inhibitory activity with an IC_50_ of 1.287 mg/mL, followed by aqueous microwave extraction (12.689 mg/mL), aqueous decoction (16.738 mg/mL), and ethanol maceration (33.942 mg/mL), respectively. Physostigmine, a reversible cholinesterase inhibitor used as a positive control, exhibited the strongest AChEI activity, with an IC_50_ of 0.826 mg/mL.

In terms of anti-inflammatory activity, the aqueous microwave extraction showed the lowest IC_50_ value of 0.3903 mg/mL, indicating the highest activity, followed by ethanol reflux extraction (1.6387 mg/mL), ethanol maceration (1.5502 mg/mL), and aqueous decoction (1.2832 mg/mL). Diclofenac, used as the positive control for anti-inflammatory activity, exhibited an IC_50_ value of 0.773 mg/mL.

### 2.6. Correlation Analysis of Phytochemical Contents and Biological Activities

**Figure 3:**
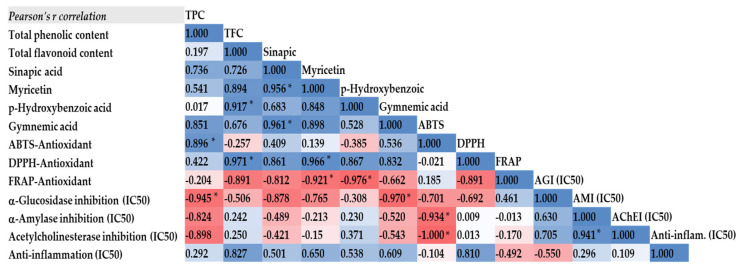
Pearson’s correlation coefficients (*r*) between the phytochemical contents, antioxidants, and anti-diabetic, anti-acetylcholinesterase, and anti-inflammatory activities. The color scale represents the strength and direction of the correlations, with blue indicating positive correlations and red indicating negative correlations. * correlation (*r*) is significant at *p* < 0.05.

A Pearson correlation coefficient analysis results showed in Figure 3 demonstrated a significant positive correlation between the total phenolic content (TPC) and ABTS scavenging activity (*r* = 0.896, *p* < 0.05), reinforcing the notion that higher phenolic content directly contributes to enhanced antioxidant activities. This correlation is particularly notable for extracts obtained via ethanolic reflux, which not only yielded higher TPC but also exhibited superior antioxidant properties. On the other hand, total flavonoid content (TFC) showed a significant positive correlation with DPPH scavenging activity (*r* = 0.971, *p* = 0.014), with myricetin, a major flavonoid, demonstrating the strongest positive correlation with DPPH scavenging (*r* = 0.966, *p* = 0.017). The antioxidant activities, particularly ABTS scavenging, tend to have a negative correlation to the half inhibitory concentration (IC_50_) to enzymatic activities (α-amylase, α-glucosidase, and acetylcholinesterase inhibition), as well as anti-inflammatory activity. These correlations indicate variations in the biological properties of the *G. inodorum* extracts across the different assays employed.

### 2.7. Cytotoxicity

**Figure 4:**
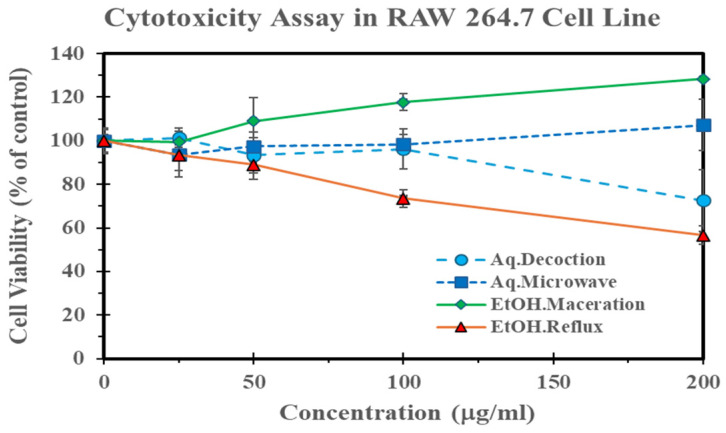
Cell viability of *G. inodorum* extracts in RAW 264.7 cells. Data are presented as the percentage of viable cells relative to the untreated control. The ethanolic reflux extract showed dose-dependent cytotoxicity, while others maintained cell viability above 80% at higher concentrations. Data represents the mean ± SD of three independent studies (*n* = 3).

A cell viability assay of *G. inodorum* extract prepared from different extraction methods in RAW 264.7 macrophage cell line demonstrated in Figure 4. Results revealed that the extracts obtained via ethanol maceration, aqueous microwave-assisted extraction, and aqueous decoction did not affect cell death at concentrations up to 100 µg/mL, with exhibited IC_50_ value of greater than 200 µg/mL. However, the ethanolic reflux extract demonstrated moderate toxicity, with an IC_50_ of 96.51 µg/mL. These results suggest that while most extracts are non-toxic at lower concentrations, the ethanolic reflux extract exhibits higher cytotoxic potential in comparison.

## 3. Discussion

The Apocynaceae family and other traditional medicinal plants have abundant phenolic compounds, including phenolics, flavonoids, phenolic acids, anthocyanins, and derivatives. Various phenolic classes have attracted significant interest due to their physiological roles, encompassing activities such as scavenging free radicals, preventing mutations, inhibiting carcinogenesis, and reducing inflammation [17,18].

The study comprehensively evaluated the efficacy of different extraction methods (aqueous decoction, aqueous microwave-assisted extraction, ethanolic maceration, and ethanolic reflux) in obtaining bioactive compounds from *Gymnema inodorum* leaves. The results highlight the significant impact of the extraction method on the extraction yield, phytochemical contents (phenolics, flavonoids, and gymnemic acid), and biological activities (antioxidant, enzyme inhibitory, anti-inflammatory activities, and cytotoxicity effect) of the extracts.

### 3.1. Effect of Extraction Methods on Extraction Yield, Phenolic and Flavonoid Content

The ethanolic reflux method, while yielding a moderate extract percentage (20.12%), resulted in the highest total phenolic content (82.54 ± 0.07 mg GAE/g) and total phenolic profile (3.605 ± 0.013 mg/g), outperforming the other extractions. Our findings showed that the phenolic content in the *G. inodorum* extracts exceeds previous reports of 20–40 mg GAE/g [19,20], highlighting its potential as a rich source of bioactive compounds. This finding can be justified by the increased temperature and solvent interaction during reflux, which enhances the breakdown of plant cell walls, facilitating the release of phenolic compounds. In contrast, the aqueous decoction method, despite producing the highest yield (28.00%), showed significantly lower phenolic content (54.88 ± 0.09 mg GAE/g), indicating that water is less effective than ethanol for extracting phenolic compounds from *G. inodorum* leaves. This observation aligns with the solvent polarity principle, where moderate polarity allows ethanol to dissolve both polar hydroxyl groups and non-polar aromatic rings in phenolics. Hence, the ability of ethanol to form hydrogen bonds with phenolic compounds enhances solubility, while its lower surface tension facilitates better penetration into plant tissues, increasing extraction efficiency [21].

The highest total flavonoid content was found in the ethanolic maceration extract (35.80 ± 0.01 mg QE/g), followed closely by the ethanolic reflux extract (31.90 ± 0.03 mg QE/g), suggesting that while both ethanol-based methods are effective for flavonoid extraction, ethanolic maceration preserves these compounds better due to lower temperatures compared to reflux extraction for such plant.

The higher extraction yield and total phenolic content observed with the ethanol reflux method can be attributed to the elevated temperature, which enhances solvent penetration and solubility, particularly for bound phenolic compounds. In contrast, the ethanol maceration method, performed at room temperature, yielded a higher total flavonoid content, likely due to the heat-sensitive nature of flavonoids which can lead to degradation during reflux extraction [22]. Hence, it indicates that ethanolic maceration is more effective at preserving flavonoids, while ethanolic reflux is better suited for extracting a broader range of phenolic compounds. These findings not only highlight the extraction efficiency but also suggest practical applications for utilizing *G. inodorum* extracts in health-related fields.

### 3.2. Effect of Extraction Methods on Gymnemic Acid Concentration

Gymnemic acid, a triterpenoid saponin, is a key bioactive compound in *Gymnema* spp., known for its anti-diabetic properties. In this study, the concentration of gymnemic acid was highest in the ethanolic reflux extract (8.24 ± 0.65 mg/g), highlighting the efficiency of this method for its extraction. Notably, this concentration far exceeds the 0.0642 ± 0.0064 mg/g gymnemic acid in air-dried *G. inodorum* leave reported in a previous study [8], further supporting the use of ethanol reflux extraction as an optimal method for extracting gymnemic acid in *G. inodorum* leaf extracts.

### 3.3. Effect of Extraction Methods on Characterisation of Phenolic Compounds

The phenolic profiles of the different extraction methods were quantified using the HPLC technique. The extraction efficiency of phenolic compounds from *G. inodorum* leaves varied significantly across different methods. Ethanol reflux extraction yielded the highest total phenolic content (3.605 ± 0.013 mg/g), demonstrating its superior ability to extract a broad range of phenolics, including sinapic acid (1.29 ± 0.01 mg/g), myricetin (0.93 ± 0.01 mg/g), p-hydroxybenzoic acid (0.22 ± 0.01 mg/g), chlorogenic acid (0.21 ± 0.01 mg/g), and (gallic acid (0.18 ± 0.01 mg/g). This suggested that high temperature in reflux extraction enhances the release of both simple and complex phenolic acids.

Ethanol maceration, although slightly lower in total phenolics (3.104 ± 0.011 mg/g), sinapic acid (0.85 ± 0.01 mg/g), and myricetin (0.72 ± 0.01 mg/g), was particularly effective for extracting compounds, such as p-hydroxybenzoic acid (0.28 ± 0.01 mg/g), p-coumaric acid (0.15 ± 0.01 mg/g), catechin (0.15 ± 0.01 mg/g), and fluoric acid (0.15 ± 0.01 mg/g), indicating that room temperature maceration preserves these heat-sensitive compounds better than reflux. The identification of p-coumaric acid and ferulic acid emphasizes the rich phytochemical profile of *G. inodorum*, with both compounds playing crucial roles in antioxidant activity and potential therapeutic applications. Their presence supports traditional uses and highlights avenues for further research into their specific health benefits.

Aqueous microwave extraction, with a total phenolic content of 1.376 ± 0.004 mg/g, was moderately efficient, particularly for sinapic acid (0.67 ± 0.01 mg/g), but less so for compounds like catechin and epicatechin, likely due to degradation at high temperatures. The aqueous decoction method produced the lowest total phenolic content (0.956 ± 0.003 mg/g) despite yielding the highest overall extract. This suggests that while water is effective in extracting bulk material, it is less efficient at solubilizing phenolics compared to ethanol-based methods. Overall, ethanol reflux was the most efficient method for phenolic extraction, while ethanol maceration was preferable for flavonoid-rich extracts.

The high efficiency of ethanol as a solvent can be attributed to its polarity, which allows it to effectively dissolve polar phenolic compounds. Ethanol and ethanol/water mixtures were found to be ideal for extracting phenolic acids because of their different polarity values and acceptability for human consumption. Waloh et al. [23] found that total phenolic and flavonoid contents were more intensive in *G. inodorum* ethanolic extracts which supports our study. The solvent’s ability to solubilize and stabilize phenolic compounds contributes to the overall higher TPC measured in ethanol extracts. Sinapic acid, myricetin, and p-hydroxybenzoic acid are generally polar due to their hydroxyl groups. Ethanol, being a polar solvent, is particularly effective in dissolving these polar compounds. The polarity of ethanol allows it to interact well with phenolic compounds, facilitating their release from the plant matrix into the solvent. As described earlier, higher temperature and heat in reflux extraction enhance the solubility of phenolic compounds in ethanol, reduce the solvent’s viscosity and surface tension, and increase kinetic energy, leading to better penetration into the plant matrix and more efficient diffusion and extraction of phenolic compounds. A study found that ethanol at 70 °C significantly improved the extraction yield of gymnemic acid from *G. inodorum* [24]. The difference in the percentage of phenolics and flavonoids present in leaf extract from *G. inodorum* was reported by another study [25]. To our knowledge, this is the first study to report the quantification of specific phenolic compounds in *G. inodorum* leaf extract, an aspect not covered in previous studies.

### 3.4. Effect of Extraction Methods on Antioxidant Activity

The antioxidative efficacy of phenolic compounds is primarily due to their redox properties, enabling them to function as reducing agents, hydrogen donors, quenchers of singlet oxygen, and potential metal chelators [26]. Such properties play a crucial role in scavenging free radicals, providing protective effects against oxidative stress-related disorders. Numerous studies have demonstrated that bioactive compounds in plant extracts possess strong antioxidant capacities, potentially promoting human health [27].

In this study, antioxidant activities were evaluated using ABTS, DPPH, and FRAP assays, where the ethanolic reflux extract exhibited the highest antioxidant activities in the ABTS (70.33 ± 0.00 mg TE/g) and DPPH (61.94 ± 0.01 mg TE/g) assays. The strong antioxidant activity correlates with the high phenolic and flavonoid content, as these compounds are known to efficiently scavenge free radicals and reduce oxidative stress.

Phenolic and flavonoid compounds are closely related, with flavonoids being a subclass of phenolic compounds, sharing similar structural characteristics and contributing to antioxidant activity. Both compounds exhibit strong hydrogen-donating abilities, which neutralize free radicals. Correlations between total phenolic content (TPC) and total flavonoid content (TFC) are often observed due to the substantial contribution of flavonoids to the overall phenolic content. However, differences in their antioxidant mechanisms, such as metal ion chelation by flavonoids, may lead to variations in correlation strength depending on the assay used.

The current study has shown that the ethanol reflux extract of *G. inodorum* contains high concentrations of phenols and flavonoids as active compounds; thus, it is inferred that these active compounds are responsible for high antioxidant capacity. The Pearson’s correlation coefficient analysis also highlighted a strong positive correlation between those compounds and antioxidant capacities. As aforementioned, we observed a strong positive correlation between the total phenolic content (TPC) and ABTS radical scavenging activity, and between the total flavonoid content (TFC) and DPPH radical scavenging activity. This indicates that phenolic and flavonoid compounds play a crucial role in antioxidant effects by donating hydrogen atoms to neutralize free radicals. The elevated radical scavenging activity observed in this study suggests that the purified compounds in the ethanol solvent system exhibit an enhanced ability to donate hydrogen atoms.

On the contrary, the aqueous decoction extract showed the highest FRAP activity (75.10 ± 0.00 mg TE/g), indicating that water-based extraction may also be effective for extracting specific antioxidant compounds. This discrepancy could be attributed to the higher concentration of free phenolic radicals in the aqueous system, which are more favorable for DPPH and ABTS reagents. Additionally, the FRAP assay measures the reduction of ferric ions to ferrous ions by electron donation, as opposed to free radical scavenging [28].

Our findings align with those of Nuchuchua et al. [9], who attributed the antioxidant activity of *G. inodorum* extract to the ability of its active compounds to transfer hydrogen atoms and electrons. This study further confirms the potent antioxidant activity of the ethanol reflux extract. However, our results also show that the aqueous decoction and maceration extracts exhibited slightly lower antioxidant activity, inconsistent with the findings of Nunta et al. [24], who reported that using 50% aqueous ethanol at 70 °C significantly enhanced antioxidant activity by increasing the yield of bioactive compounds. These results suggest that the impact of the extraction method extends beyond the choice of solvent, highlighting the importance of other factors, such as temperature and extraction conditions, in influencing antioxidant activity.

While the high antioxidant capacity of *G. inodorum* extract is generally attributed to its high phenolic content, our results indicate that aqueous extracts with lower TPC also exhibited significant antioxidant activity. This suggests that in addition to phenolic compounds, other factors such as extraction temperature and solvent type may also play a crucial role in contributing to an extract’s overall antioxidant activity.

### 3.5. Effect of Extraction Methods on Alpha-Glucosidase and Alpha-Amylase Inhibitory Activities

The ethanolic reflux extract demonstrated the most potent α-glucosidase inhibition (IC_50_ = 7.3976 mg/mL) and significant α-amylase inhibition (IC_50_ = 13.36 mg/mL), indicating a strong potential for managing postprandial hyperglycemia, a key aspect of diabetes management. The lower IC_50_ values reflect higher potency compared to the other extracts, suggesting that even at relatively low concentrations, the ethanolic reflux extract can effectively inhibit the enzymes involved in carbohydrate digestion, making it suitable for anti-diabetic applications. These findings are consistent with previous studies on *G. inodorum,* which have reported notable α-glucosidase and α-amylase inhibitory effects, reinforcing its potential in diabetes management [29,30].

The dual inhibitory activity of the ethanolic reflux extract provides a comprehensive approach to controlling blood glucose levels, targeting both the early and later stages of diabetes. The potent anti-diabetic properties of the ethanolic reflux extract observed in this study seem to be associated with the concentration of bioactive compounds, such as gymnemic acid. This is demonstrated by the negative correlations between gymnemic acid concentration and the IC_50_ values for α-glucosidase (*r* = −0.970, *p* = 0.015), and α-amylase (*r* = −0.520, *p* = 0.240). Specifically, as gymnemic acid concentration increases, the IC_50_ value decreases, indicating enhanced enzyme inhibition and suggesting a stronger anti-diabetic potential. Additionally, significant correlations were observed between α-glucosidase inhibition and the total phenolic content (*r* = −0.945, *p* = 0.028). These findings suggested that the phenolic compounds in *G. inodorum* leaf extracts may also contribute to its anti-diabetic activity, particularly through a mechanism involving the inhibition of the α-glucosidase pathway.

### 3.6. Effect of Extraction Methods on Acetylcholinesterase Inhibition (AChEI) and Anti-Inflammatory Activity

The increased potency observed in AChE inhibitory (AChEI) activity is likely attributable to the elevated total phenolic content (*r* = −0.898, *p* = 0.051), while the anti-inflammatory activity is more likely associated with the total flavonoid content (*r* = 0.827, *p* = 0.087) in the extract.

Flavonoids, a subgroup of polyphenols, are ubiquitous in plant tissues and are quintessential components of many plant-based foods [31]. They include compounds such as caffeic acid, catechin, chlorogenic acid, coumarin, quercetin, rutin, and kaempferol. While flavonoids are primarily noted for their anti-inflammatory properties [32], some studies indicate they may also exhibit pro-inflammatory effects under specific conditions [33]. High concentrations or certain structural forms may increase cytokine production (e.g., IL-6, TNF-α) in immune cells, potentially exacerbating inflammation in conditions like inflammatory bowel disease (IBD) depending on concentration and the inflammatory environment.

Research has shown that flavonoids like quercetin, catechin, and their derivatives exhibit both anti-inflammatory and anti-diabetic properties [34]. These findings provide a scientific basis for the extensive historical use of *G. inodorum* in traditional folk medicine and offer evidence supporting its potent bioactive effects.

### 3.7. Effect of Extraction Methods on Cytotoxicity of G. inodorum Extracts

The cytotoxicity of the *G. inodorum* extracts was evaluated using the MTT assay on RAW264.7 macrophage cells. The cells were treated with various concentrations of the *G. inodorum* extracts for 24 h. The ethanolic reflux extract demonstrated moderate cytotoxicity, with an IC_50_ value of 96.51 µg/mL. In contrast, the ethanolic maceration extract showed no cytotoxic effect on cell viability. These findings are consistent with a previous report which reported low cytotoxicity for *G. inodorum* ethanolic maceration extract, showing an IC_50_ of 128.77 ± 2.82 µg/mL against RAW264.7 cells [25]. This suggests that the method of extraction influences the cytotoxicity of the extracts. These results, however, suggest that ethanolic *G. inodorum* leaf extract may have a relatively mild effect on cell viability at the tested concentrations.

Taking together, this study demonstrates the ability of the active compounds in the ethanol reflux extract to effectively scavenge free radicals, suggesting the presence of specific phenolic compounds that may serve as therapeutic agents in mitigating radical-induced pathological damage. These findings highlight the potential safety of the plant for human use and contribute to a deeper understanding of the therapeutic window and safety profile of the extract. The mild-to-moderate cytotoxicity observed indicates that while the extract possesses potent bioactive properties, its application should be carefully managed to prevent potential adverse effects.

## 4. Materials and Methods

### 4.1. Chemicals and Regents

Analytical grade (AR) solvents, including methanol, chloroform, acetonitrile, and the gymnemic acid standard, deacylgymnemic acid (CAS Number: 121686-42-8) were pro-cured from Sigma-Aldrich (St. Louis, MO, USA).

### 4.2. Plant Material and Extraction Methods

The shoot and the first three pairs of *G. inodorum* leaves were harvested from Sanmahaphon Organic Herbs Community Enterprise, Chiang da Garden, Chiang Mai, Thailand. Subsequently, the leaves were meticulously purified and air-dried. The dried leaves were ground and sieved through a sieve shaker using a 60-mesh sieve size to yield a particle size of 250 μm. Ten grams of powdered leaves were then subjected to extraction through different methods repeated in triplicate to obtain a crude extract. The extraction methods included aqueous decoction (20 min boiling), microwave-assisted extraction (800 W for 5 min), ethanolic maceration (24 h at room temperature), and ethanolic reflux extraction (80 °C for 120 min). These parameters were chosen based on the literature indicating optimal conditions for phenolic compound extraction [35,36] and refined through preliminary trials. The extract was concentrated using a rotary evaporator and lyophilized. The yield was calculated as a percentage for further analysis using the formula:

yield (%) = mass of crude extract (g)/mass of sample (g) × 100

#### 4.2.1. Aqueous Decoction

The plant material was dried in a hot air oven at 50 °C and then ground into powder using an electric blender. The dried powder was used to prepare the extract through a hot water extraction method. A total of 10 g of the dried leaves was soaked in 100 mL of distilled water and heated in a water bath at 80 °C for 20 min. The filtrate was then obtained through vacuum filtration using Whatman No. 1 filter paper (Cytiva, Little Chalfont, Buckinghamshire, UK), and the liquid was concentrated through lyophilization. The dried powder form of the aqueous crude extract of *G. inodorum* was stored at −20 °C for future use [37].

#### 4.2.2. Microwave-Assisted Aqueous Extraction

Microwave digestion was performed using an ETHOS UP system (Milestone Srl, Sorisole, Italy), equipped with a dual 950-watt magnetron configuration (total power: 1900 W) and a 70-L stainless steel cavity for high-throughput sample preparation. The blended powder of *G. inodorum* was added into tubes of auto microwave extractor in 1:10 (*w*/*v*). A total of 3 g powder was added to 30 mL distilled water and placed in the extractor for 5 min on 800 W, 60 °C plus 10 min for self-washing. The resulting extract was collected and filtered through vacuum filtration using Whatman No. 1 filter paper (Cytiva, Little Chalfont, Buckinghamshire, UK), and then the crude extract was subjected to dried using a Christ Alpha 1-4 LSCplus freeze dryer (Martin Christ Gefriertrocknungsanlagen GmbH, Osterode am Harz, Germany) equipped with a condenser temperature of −55 °C and a condenser capacity of 4 kg. Dry sample was weighed and stored at a temperature of −20 °C for further bioassays.

#### 4.2.3. Ethanol Maceration

The dried leaves of *G. inodorum* were ground in an electronic blender into a fine powder. A total of 10 g of the sample was macerated with 100 mL (95%) ethanol in (1:10 *w*/*v*) for 1 day at room temperature. The plant extract was filtered using Whatman No. 1 filter paper (Cytiva, Little Chalfont, Buckinghamshire, UK), and then concentrated into a crude extract form using a rotary evaporator (Model RV 10, IKA, Staufen, Germany). This crude extract was subjected to freeze-drying equipment mention earlier, weighed and stored at −20 °C for further analysis.

#### 4.2.4. Ethanol Reflux Extraction

The dried leaves were finely powdered, and 10 g of this powdered material from *G. inodorum* was packed into a cellulose thimble. Next, a 95 percent ethanol solution (100 mL) was added to the thimble at a ratio of 1:10 (*w*/*v*). The extraction process was performed thrice at 80 °C using an automatic automatic FatExtractor E-500 system (BÜCHI Labortechnik AG, Flawil, Switzerland), each running for 2 h. After this extraction, the resulting solution was subjected to evaporation using a rotary evaporator to yield the crude extract. Subsequently, the extract was desiccated in a chemical hood, reweighed, and stored at 4 °C for further analysis.

### 4.3. Phytochemical Analysis

#### 4.3.1. Total Phenolic Content

A quantitative analysis of phenolic content was conducted using the modified Folin–Ciocalteu colorimetric method, using gallic acid as the standard reference. To perform the analysis, a mixture of 20 µL of *G. inodorum* extract and 20 µL of the diluted (10 times in distilled water) Folin–Ciocalteu solution was prepared. The mixture was then combined with 80 µL of 7% Na_2_CO_3_ and 200 µL of DW. The plate was observed for any turbidity, and immediately at 760 nm, the absorbance was recorded by spectrophotometer (FLUOstar^®^ Omega, BMG LABTECH GmbH, Ortenberg, Germany) to calculate the total phenolic content. The findings were expressed in mg of gallic acid equivalent (mg GAE/g sample).

#### 4.3.2. Quantification of Phenolic Profile

For the analysis of phenolic compounds, a High-Performance Liquid Chromatography (HPLC) system (Shimadzu, Kyoto, Japan) equipped with an SPD-M20A Prominence Diode Array Detector (DAD) was utilized. An Inertsil C18 column (250 × 4.6 mm, GL Sciences, Torrance, CA, USA) served as the stationary phase. The mobile phases consisted of mobile phase A (2% acetic acid in water) and mobile phase B (100% acetonitrile). The methodology for determining phenolic compounds was described elsewhere [38]. Briefly, approximately 1 mL of shiitake extract was mixed with 0.5 mL of Folin–Ciocalteu reagent, followed by 3 mL of 20% Na_2_CO_3_ solution. After 30 min of reaction, the mixture was diluted with 10 mL of deionized water and incubated for 15 min. The absorbance was measured at 760 nm, and the total phenolic content was expressed as milligrams of gallic acid equivalent per 100 g dry weight. For the phenolic compound analysis, HPLC (Shimadzu, Kyoto, Japan) with an SPD-M20A Diode Array Detector (DAD) and an Inertsil C18 column (250 × 4.6 mm) was used. A gradient elution with mobile phase A (2% acetic acid in water) and mobile phase B (100% acetonitrile) was employed. The flow rate was 1 mL/min, with the column temperature set at 30 °C. Individual *G. inodorum* extract was diluted with acetonitrile, filtered through a 0.45 µm membrane, and 10 µL was injected for analysis. To ensure the accurate identification and quantification of phenolic compounds, nineteen phenolic standards were used as references, including (1) gallic acid, (2) Theobromine, (3) Protocatechuic acid, (4) p-Hydroxybenzoic acid, (5) catechin, (6) chlorogenic acid, (7) Caffeine, (8) Vanillic acid, (9) caffeic acid, (10) Syringic acid, (11) epicatechin, (12) Vanillin, (13) p-Coumaric acid, (14) ferulic acid, (15) sinapic acid, (16) rutin, (17) myricetin, (18) quercetin, and (19) Trans-cinnamic acid. These standards were detected at 280 nm, with their retention times and UV spectra compared with those of the compounds in the *G. inodorum* extracts. The quantification was performed using external calibration curves prepared with the authentic standards, and the results were expressed as mg of compound per g of extract.

#### 4.3.3. Total Flavonoid Content

The total flavonoid content was determined by using quercetin as the standard through colorimetric aluminum chloride assays. To perform this, 25 µL of the sample, blank, or various concentrations of standard solution were added to a 96-well microtiter plate with 12.5 µL of DW and 7.5 µL of 7% NaNO_3_ solution. The mixture was allowed to sit at ambient temperature for 5 min. Afterwards, 10% 15 µL of AlCl_3_ solution was added and thoroughly mixed. The plate was stored for another 5 min at room temperature. Finally, 27.5 µL of distilled water and 50 µL of 1 M NaOH solution were added and incubated at room temperature for an additional 5 min. At 510 nm, the absorbance was measured using water as a reference, and the results were reported in terms of the quercetin equivalent per gram of sample (mg QE/g sample).

#### 4.3.4. Gymnemic Acid Content

For the detection of gymnemic acid, 5 mg of the crude gymnema extract was dissolved in 10 mL of a 50% (*v*/*v*) methanol solution for chromatographic analysis. This solution, after adding 1 mL of 11% KOH, was heated under reflux for one hour in a boiling water bath before being cooled. After that, 0.9 mL of 12 N hydrochloric acid (HCl) was mixed and refluxed for another hour in the water bath. After lowering the temperature, the solution was mixed with 10% (*v*/*v*) methanol and further subjected to analyzation with HPLC (Shimadzu LC, Kyoto, Japan). The concentration of gymnemic acid was calculated using the sample’s area under the curve, and it was compared to the standard value of deacyl gymnemic acid. Chromatography was conducted using LC systems, HPLC with analytical C18 column (250 × 4.60 mm i.d. × 5.0 μm), mobile phase consisting of (A) acetonitrile solution and (B) type 1 water (50:50) at 30 °C column temperature, a flow rate of 2.0 mL/min, and detected at the wavelength of 218 nm. Deacyl gymnemic acid was used as a standardization concentration (3.91–500 μg/mL), and a duplicate sample was injected along with 5 μL of the sample solution to perform the HPLC estimation [39].

### 4.4. Antioxidant Analysis

The *G. inodorum* leaf extracts were evaluated for their potential antioxidant capacity as well as free radical scavenging activities using 3 standard antioxidant assays previously described [40].

#### 4.4.1. DPPH Radical Scavenging Activity

The DPPH assay was used to assess the free radical scavenging capabilities of the *G. inodorum* extracts. The procedure involved creating a DPPH stock solution in methanol and adding 195 µL of the DPPH solution with 10 µL of *G. inodorum* extract in a 96-well microtiter plate. After a 30 min dark incubation period, the absorbance at 517 nm was obtained using a SPECTROstar nano microplate reader (BMG LABTECH GmbH, Ortenberg, Germany).

% scavenging activity = [(A_0_ − A_1_)A_0_] × 100%

where A_0_ = absorbance of control and A_1_ = absorbance of the extract with DPPH

#### 4.4.2. ABTS Decolorization Assay

The ABTS decolorization assay was employed to evaluate the antioxidant potential of the *G. inodorum* extracts. The ABTS reagent was prepared by mixing 7 mM 2,2′-azino-bis (3-ethylbenzthiazoline-6-sulphonic acid) with 2.45 mM potassium persulfate (K_2_S_2_O_8_) in a 1:1 (*v*/*v*) ratio. This mixture was allowed to react in the dark for 12 h at room temperature. After incubation, the ABTS solution was diluted with 80% ethanol to the desired concentration. In a 96-well microtiter plate, 10 µL of the *G. inodorum* extract at various concentrations was added, with controls prepared without the extract. After a 30 min incubation in the dark, the absorbance was measured at a wavelength of 734 nm. The results were expressed as the 50% inhibition concentration (IC_50_), reflecting the correlation between the % inhibition and the concentration of the test samples. The inhibition percentage was calculated in comparison to Trolox, used as the standard reference antioxidant.

#### 4.4.3. FRAP Assay

The ferric-reducing antioxidant power (FRAP) of the *G. inodorum* extract was determined using a modified colorimetric assay. The FRAP reagent was prepared by combining 2.5 mL of 10 mM 2,4,6-tripyridyl-s-triazine (TPTZ) in 40 mM HCl, 2.5 mL of 20 mM ferric chloride (FeCl_3_), and 25 mL of 300 mM acetate buffer (pH 3.6). The *G. inodorum* extract was diluted twofold with deionized water, and 10 µL of the diluted extract was added to 190 µL of the FRAP reagent in a 96-well microtiter plate. Following a 30 min incubation in the dark, the absorbance was measured at 593 nm using a SPECTROstar Nano microplate reader. The FRAP value was calculated by comparison with Trolox, used as the standard reference.

### 4.5. α-Glucosidase and α-Amylase Enzyme Inhibitory Activity

The preliminary screening of the *G. inodorum* extracts for enzyme inhibition activity against α-glucosidase and α-amylase, along with the reference compound acarbose, was conducted following a previously described method [41] with slight modifications. Varying concentrations of the extracts (0.001–10 mg/mL) were prepared in DMSO.

Briefly, 100 μL of the test substance was mixed with 200 μL of α-amylase and 100 μL of 2 mM phosphate buffer (pH 6.9) containing 0.02 M sodium phosphate (pH 6.9). After incubation for 20 min, 100 μL of a 1% starch substrate was added. Blank controls, where the enzyme was replaced with the buffer, were also prepared. Following a 5 min incubation of the reaction mixture, 500 μL of DNSA reagent was added to both the control and test samples. These mixtures were then heated in a boiling water bath for 5 min. Absorbance readings were taken at 540 nm, and the percentage inhibition was calculated using the following formula:

% inhibition = (Abs_control_ − Abs_sample_) Abs_control_ × 100

where Abs_control_ = absorbance of the blank control (containing all the reagents including 20% DMSO except the sample solution) and Abs_sample_ = absorbance of the test sample. The half-maximal inhibitory concentration (IC_50_) values were determined and expressed as IC_50_ values.

An assay for α-glucosidase inhibitory activity was conducted to determine the anti-diabetic activity of *G. inodorum* extract. Briefly, 60 μL of the sample solution and 50 μL of 0.1 M phosphate buffer containing α-glucosidase solution (0.2 U/mL) were incubated in a 96-well plate at 37 °C for 20 min. After pre-incubation, 50 μL of the substrate solution (5 mM p-nitrophenyl-α-D-glucopyranoside (p-NPG) in 0.1 M phosphate buffer, pH 6.8) was added to each well and further incubated at 37 °C for another 20 min. To terminate the reaction, 160 μL of 0.2 M Na_2_CO_3_ was added to each well. The absorbance was measured at 405 nm using a microplate reader and compared to the control, which contained 60 μL of buffer solution instead of the extract. For blank incubation (to account for absorbance produced by the extract), the enzyme solution was replaced with the buffer solution, and absorbance was recorded. Acarbose, a known α-glucosidase inhibitor, was used as a positive control. The percentage inhibition of α-glucosidase activity was calculated using the following equation:

% inhibition = [(Abs_blank_ − Abs_sample_)/Abs_blank_] × 100

where Abs_blank_ is the absorbance of the control without the sample solution, and Abs_sample_ is the absorbance of the sample with the extract solution. IC_50_ value, the concentration of the *G. inodorum* extract required to inhibit 50% of α-glucosidase activity, was determined and compared to Acarbose.

### 4.6. Inhibitory Activities to Acetylcholinesterase and Inflammation

The AChEI activity assay was performed using the Acetylcholinesterase Inhibitor Screening Kit (Sigma-Aldrich, USA) according to the manufacturer’s protocol. The assay is based on the improved Ellman method, which measures the ability of the test sample to inhibit acetylcholinesterase activity. In this method, thiocholine, produced by the action of acetylcholinesterase, reacts with 5,5′-dithiobis(2-nitrobenzoic acid) to form a yellow-colored product. The intensity of this color, measured at 412 nm, is proportional to the enzyme activity in the sample. Physostigmine, a known acetylcholinesterase inhibitor, was used as a positive control.

The anti-inflammatory activity was evaluated for its influence on the production of nitric oxide. The anti-inflammatory activity of the *G. inodorum* extract was evaluated using a colorimetric assay. The extract was dissolved in DMSO to achieve concentrations of 10 µg/mL, 50 µg/mL, and 100 µg/mL. In a 96-well microplate, 50 µL of each extract concentration and diclofenac (used as a positive control) were added to separate wells, followed by the addition of 50 µL of Griess reagent. The plate was incubated at room temperature for 10 min for color development. Absorbance was then measured at 540 nm using a microplate reader. The percentage inhibition of nitric oxide (NO) production was calculated using the absorbance values of the untreated samples and diclofenac-treated wells as controls.

### 4.7. Cytotoxicity of GI Extract

The RAW 264.7 macrophage cells were obtained from the American Type Culture Collection (ATCC^®^ TIB-71™, Manassas, VA, USA). These cells were cultured in Dulbecco’s Modified Eagle Medium (DMEM, ATCC^®^ 30-2002™, Manassas, VA, USA) supplemented with 10% fetal bovine serum and 1% penicillin–streptomycin antibiotics, maintained at 37 °C in a 5% CO_2_ incubator. The cells were seeded at a density of 2.5 × 10^5^ cells/mL in 25 cm^3^ flasks and incubated under the same conditions. After 24 h, the medium was discarded, and the cells were rinsed twice with PBS. DMEM (2–3 mL) was then added to each flask. The cells were subsequently counted and diluted to a density of 2.5 × 10^5^ cells/mL in a 96-well plate for cell viability assays.

Cell viability was assessed using the MTT assay, which is based on the reduction of MTT to formazan by metabolically active cells. This study utilized the MTT assay to determine the viability of RAW 264.7 cells following treatment with various concentrations (25, 50, 100, 200, 400, and 800 μg/mL) of the *G. inodorum* leaf extract. The RAW 264.7 cells were cultured in 96-well plates containing DMEM and incubated overnight at 37 °C in a 5% CO_2_ incubator for 24 h. After incubation, the medium was removed, and the cells were washed with PBS. *G. inodorum* leaf extract solutions, prepared by dissolving the extract in dimethyl sulfoxide (DMSO) to achieve a final DMSO concentration of <0.1% (*v*/*v*), were added to each well along with LPS (1 μg/mL), a positive control (10% DMSO), and a negative control (blank) to a total volume of 100 μL per well. The plates were incubated at 37 °C in a 5% CO_2_ incubator for an additional 24 h. Following treatment, the medium was removed, and 15 μL/well of 5 mg/mL MTT solution in PBS, along with 100 μL/well of fresh culture medium, was added. The plates were incubated for 24 h at 37 °C. After incubation, the medium was discarded, and 100 μL of DMSO was added to each well to dissolve the formazan crystals by gentle shaking for 15 min. Absorbance was measured at 450–620 nm using a SPECTROstar Nano microplate reader (BMG LABTECH) to quantify cell viability. The cytotoxicity of the *G. inodorum* extract was expressed as the IC_50_ value, defined as the concentration of the extract required to reduce cell viability by 50%.

### 4.8. Statistical Analysis

The data were analyzed using a one-way analysis of variance (ANOVA), followed by Duncan’s multiple range test to compare the mean values, using SPSS version 23. All the results are expressed as mean ± standard deviation (n = 3). A Pearson correlation analysis was also conducted to evaluate the relationships between the various phytochemical parameters (total phenolics and total flavonoids), antioxidant activities (ABTS, DPPH, and FRAP), enzyme inhibitory activities (α-glucosidase, α-amylase, and acetylcholinesterase), and anti-inflammatory activity.

## 5. Conclusions

In conclusion, the ethanolic reflux extraction method has been demonstrated as the most effective technique for extracting bioactive compounds from *G. inodorum*. This method yields significant antioxidants, enzyme inhibition, and anti-inflammatory activities, along with moderate cytotoxicity. The successful extraction of key phenolic compounds highlights the phytochemical richness of *G. inodorum* and underscores the importance of selecting optimal extraction methods to maximize therapeutic potential, thus fulfilling the research objective of identifying the most effective technique for enhancing the pharmacological profile of *G. inodorum* as outlined in the introduction. The study revealed the extraction of the main phenolic compounds in the ethanol reflux extract of *G. inodorum*. Hence, *G. inodorum* possesses a significant phytochemical compound and exhibits beneficial pharmacological properties. Our findings suggest promising therapeutic applications for *G. inodorum* extracts in managing oxidative stress-related disorders, diabetes, and inflammation. However, the study acknowledges limitations due to its reliance on in vitro studies, indicating a need for further validation through in vivo experiments. Future research should prioritize clinical trials to explore the clinical relevance of the identified bioactive compounds and investigate their synergistic effects, enhancing our understanding of their therapeutic potential. Ultimately, this research contributes to our knowledge of the medicinal properties of *G. inodorum* and encourages further exploration into its therapeutic applications, with careful consideration required for dosage and formulation to ensure the safe and effective use of its extracts.

## Figures and Tables

**Table 1:** The extraction yield and phytochemical content of *G. inodorum* leaf extract.

Extraction Method	Yield (%)	Total Phenolic Content (mg GAE/g)	Total Flavonoid Content (mg QE/g)	Gymnemic Acid Content (mg/g)
Aq. Decoction	28.00	54.88 ± 0.09 ^a^	12.2 ± 0.07 ^a^	5.51 ± 0.86 ^a^
Aq. Microwave	27.18	55.49 ± 0.22 ^a^	8.12 ± 0.00 ^b^	5.61 ± 0.82 ^a^
EtOH. Maceration	7.58	45.21 ± 0.12 ^b^	35.80 ± 0.01 ^c^	6.24 ± 0.57 ^b^
EtOH. Reflux	20.12	82.54 ± 0.07 ^c^	31.90 ± 0.03 ^d^	8.24 ± 0.65 ^c^

Total phenolic content was quantified using the Folin–Ciocalteu colorimetric assay and is reported as milligrams of gallic acid equivalents (mg GAE) per gram of extract. Total flavonoid content was measured using the aluminum chloride colorimetric method and is expressed as milligrams of quercetin equivalents (mg QE) per gram of extract. Gymnemic acid concentration was determined using HPLC with authentic standards and reported as milligrams per gram of extract. The statistical significance was determined using one-way ANOVA, followed by Duncan’s multiple range test at *p* < 0.05 and represented by lowercase letters (a, b, c, or d). The variables with the same letters are not statistically significant. Data are expressed as mean ± SD (*n* = 3).

**Table 2:** Phenolic profile and composition of extracts of *G. inodorum*.

No	Phenolic Compounds (mg/g)	RT (min)	Extraction Methods			
			Aq. Decoction	Aq. Microwave	EtOH. Maceration	EtOH. Reflux
1	Gallic acid	6.38	ND	ND	0.02 ± 0.01	0.18 ± 0.01
2	Theobromine	10.07	ND	ND	0.04 ± 0.01	ND
3	Protocatechinic acid	11.45	ND	ND	0.06 ± 0.01	0.04 ± 0.01
4	p-Hydroxybenzoic acid	18.63	0.09 ± 0.01	0.14 ± 0.01	0.28 ± 0.01	0.22 ± 0.01
5	Catechin	20.21	0.03 ± 0.01	0.04 ± 0.01	0.15 ± 0.01	0.11 ± 0.01
6	Chlorogenic acid	23.00	0.07 ± 0.01	0.01 ± 0.01	0.11 ± 0.01	0.21 ± 0.01
7	Caffeine	24.12	ND	ND	0.01 ± 0.01	ND
8	Vanillic acid	24.98	ND	ND	0.03 ± 0.01	ND
9	Caffeic acid	26.76	0.02 ± 0.01	0.02 ± 0.01	0.06 ± 0.01	0.03 ± 0.01
10	Syringic acid	30.15	0.02 ± 0.01	0.02 ± 0.01	0.02 ± 0.01	0.03 ± 0.01
11	Epicatechin	33.97	ND	ND	0.05 ± 0.01	ND
12	Vanillin	35.26	0.03 ± 0.01	0.03 ± 0.01	0.07 ± 0.01	0.05 ± 0.01
13	p-Coumaric acid	40.46	0.03 ± 0.01	0.03 ± 0.01	0.25 ± 0.01	0.07 ± 0.01
14	Ferulic acid	47.18	0.03 ± 0.01	0.04 ± 0.01	0.15 ± 0.01	0.09 ± 0.01
15	Sinapic acid	49.06	0.44 ± 0.01	0.67 ± 0.01	0.85 ± 0.01	1.29 ± 0.01
16	Rutin	55.73	0.01 ± 0.01	0.06 ± 0.01	0.05 ± 0.01	0.13 ± 0.01
17	Myricetin	60.12	0.20 ± 0.01	0.33 ± 0.01	0.72 ± 0.01	0.93 ± 0.01
18	Quercetin	65.12	ND	0.01 ± 0.01	0.08 ± 0.01	0.14 ± 0.01
19	Trans-cinnamic acid	65.57	0.02 ± 0.01	0.02 ± 0.01	0.10 ± 0.01	0.08 ± 0.01
	Summary		0.986 ± 0.003 ^a^	1.426 ± 0.004 ^a^	3.104 ± 0.011 ^b^	3.605 ± 0.013 ^c^

HPLC result of phenolic profile. Compounds were identified based on their retention times and characteristic UV absorbance spectra compared to those of authentic standards. The quantification of components was performed by comparison of peak area and expressed as mg/g of extract. The variables with similar lowercase letters (a, b, or c) are not statistically significant (*p* > 0.05). Each value represents the mean ± SD (*n* = 3); ND = not detected.

**Table 3:** Alpha-glucosidase inhibitory (AGI) and alpha-amylase inhibitory (AMI) activities of *G. inodorum* leaf extracts prepared from different extraction methods.

Extraction Method	AGI (IC_50_, mg/mL)	AMI (IC_50_, mg/mL)
Aq. Decoction	112.587	13.231
Aq. Microwave	39.086	13.666
EtOH. Maceration	158.371	13.273
EtOH. Reflux	13.362	7.398
Acarbose (Positive control)	5.534	0.999

Data are expressed as a half maximal inhibitory concentration (IC_50_). Acarbose, an anti-diabetic drug, was used as a positive control. All the measurements were performed in triplicate.

**Table 4:** Acetylcholinesterase inhibitory (AChEI) and anti-inflammatory activities of *G. inodorum* leaf extracts prepared from different extraction methods.

Extraction Method	AChEI (IC_50_, mg/mL)	Anti-Inflammatory (IC_50_, mg/mL)
Aq. Decoction	16.738	1.2832
Aq. Microwave	12.689	0.3903
EtOH. Maceration	33.942	1.5502
EtOH. Reflux	1.287	1.6387
Physostigmine (Positive control)	0.826	N/A
Dichlofinac (Positive control)	N/A	0.773

Data are expressed as a half maximal inhibitory concentration (IC_50_). Physostigmine, an acetylcholinesterase inhibitor, and Dichlofinac, a nonsteroidal anti-inflammatory drug, were used as positive controls. All the measurements were performed in triplicate. N/A: not assessed.

## Data Availability

Data are contained within the article.
